# Small hard drusen and associated factors in early seniority

**DOI:** 10.1371/journal.pone.0279279

**Published:** 2022-12-22

**Authors:** Mohamed Belmouhand, Simon P. Rothenbuehler, Sami Dabbah, Jakob Bjerager, Birgit Sander, Jacob B. Hjelmborg, Christine Dalgård, Rasmus Jensen, Michael Larsen

**Affiliations:** 1 Department of Ophthalmology, Rigshospitalet, Copenhagen University Hospital, Glostrup, Denmark; 2 Faculty of Health and Medical Science, Department of Clinical Medicine, University of Copenhagen, Copenhagen, Denmark; 3 Department of Ophthalmology, University Hospital Basel, Basel, Switzerland; 4 Epidemiology, Biostatistics and Biodemography, Department of Public Health, University of Southern Denmark, Odense, Denmark; 5 Danish Twin Research Center, University of Southern Denmark, Odense, Denmark; 6 Clinical Pharmacology, Pharmacy and Environmental Medicine, Department of Public Health, University of Southern Denmark, Odense, Denmark; 7 Department of Mathematics and Computer Science, University of Southern Denmark, Odense, Denmark; University of Rochester FEI: University of Rochester David and Ilene Flaum Eye Institute, UNITED STATES

## Abstract

**Purpose:**

The purpose of this study was to examine the ocular and systemic risk profile of the fundus phenotype ≥ 20 small hard (macular) drusen (< 63 μm in diameter).

**Methods:**

This single-center, cross-sectional study of 176 same-sex twin pairs aged 30 to 80 (median 60) years was a component of a framework study of the transition from not having age-related macular degeneration to having early AMD. Drusen categories assessed using fundus photography and optical coherence tomography included small hard drusen (diameter < 63 μm), intermediate soft drusen (63–125 μm), and large soft drusen (> 125 μm), of which the soft drusen are compatible with a diagnosis of AMD.

**Results:**

Having ≥ 20 small hard drusen within or outside the macula was associated with increasing age, lower body mass index, shorter axial length, hyperopia, female sex, increasing high-density lipoprotein (HDL), high alcohol consumption, and with the presence of soft drusen.

**Conclusions:**

Having ≥ 20 small hard drusen was associated with some AMD-related risk factors, but not with smoking, increasing body mass index, and higher blood pressure. Having ≥ 20 small hard drusen was also associated with soft drusen, in agreement with previous studies. These findings suggest that small hard drusen are not an early manifestation of AMD but the product of a distinct process of tissue alteration that promotes the development of AMD or some subtype thereof.

## Introduction

Small hard drusen are sharply demarcated bright elements located within or behind the retinal pigment epithelium. They typically have a diameter smaller than 63 μm [1–3]. Our study group showed in a recently published 20-year follow-up twin study that ≥ 20 small hard drusen is significantly associated with drusen ≥ 63 μm [4], which is the conventional threshold for diagnosing age-related macular degeneration (AMD). In the Watermen Study, the presence of numerous (≥ 5) small hard drusen was associated with a higher incidence of soft drusen ≥ 63 μm [5]. The association between small hard drusen and soft drusen ≥ 63 μm was confirmed by the Beaver Dam Eye Study [6]. It is unknown if small hard drusen are direct topographic precursors of soft drusen or if soft drusen develop independently of the exact location of small hard drusen. However, the empirical risk factors of small hard drusen are obviously of interest for the study of AMD.

The risk of having ≥ 20 small hard drusen per eye was previously examined in the Inter99 cohort [7]. It was associated with moderately elevated triglycerides and low serum high-density lipoprotein (HDL) in a study population with a median age lower than 50 years and relatively few cases with AMD.

Established risk factors for AMD include age [8], tobacco smoking [8], genetic predisposition [9,10], race [11], cardiovascular disease [Age-Related Eye Disease Study Research Group 12], relative hyperopia [13], shorter axial length [14], fatty foods [15,16] and a higher body mass index [17].

The present study aimed to examine risk factors for small hard drusen in comparison with known AMD-associated risk factors in a cohort of twins with a median age higher than 50 years.

## Materials and methods

The observational Copenhagen Twin Cohort Eye Study includes pairs of twins from the Danish Twin Registry [18] in a balanced proportion of monozygotic and dizygotic pairs. The study is designed to examine the interaction between genes and environment in the development of eye and vision characteristics. A subgroup of the participants first underwent an eye examination in 1999 as part of a longitudinal cohort study (GEMINAKAR [19,20]). Additional participants with a comparable sex and zygosity distribution, but a wider age span, were recruited in 2019 from the Danish Twin Registry. Oral and written informed consent was obtained from the participants. Ethics Committee approval was obtained (registration number H-18052822) along with Danish Data Protection Agency approval (registration number VD-2018-434). The study adhered to the tenets of the Declaration of Helsinki.

The study included same-sex pairs of twins 30 years or older. The study excluded pairs of twins in whom one or both had an ocular disorder that interfered with retinal imaging. Participants were enrolled between March 1, 2019 and June 31, 2020.

Past medical history included all current and previous diseases, including non-ocular diseases, family history of eye diseases, contact lens use, current medication and allergies. The following parameters were recorded at the study visit: Sex, zygosity, ethnicity, height, weight, alcohol consumption, and smoking. Smoking was defined as active/history tobacco use if ≥ 1 pack-year (≥ 20 cigarettes daily for 365 consecutive days). Alcohol consumption was categorized as below or above 14 units/week for men or 7 units/week for women (1 unit = 12 g ethanol). Because few participants had hypertension, we used mean arterial pressure to assess the role of blood pressure. Quantification of smoking, alcohol consumption, height, and weight was self-reported. Because the number of participants who have diabetes was low, the assessment of glucose metabolism was based on glycated hemoglobin (HbA_1c_).

A blood sample was drawn from each participant and analyzed for glycated hemoglobin (HbA_1c_), triglycerides, low-density lipoprotein (LDL), high-density lipoprotein (HDL), very low-density lipoprotein (VLDL), and total cholesterol.

Participants underwent a complete ophthalmic examination including assessment of refraction and best-corrected visual acuity (BCVA) using Early Treatment Diabetic Retinopathy Study (ETDRS) charts at four meters, slit lamp examination, rebound tonometry, mydriatic fundus photography, spectral-domain optical coherence tomography (SD-OCT; Spectralis OCT 2, Heidelberg Engineering, Heidelberg, Germany) in the form of disc topography (25 disc-centered, radial b-scans and three circumpapillary scans in high resolution), and a macula volume scan (20x20 degree, 97 b-scans, high resolution and 20-scan averaging). Mydriatic color and red-free fundus photography (Topcon, TRC 50DX, Tokyo, Japan) included one color image (50° fovea-centered image) and four red-free images (one fovea-centered, one disc-centered, one superior arc-centered, and one inferior arc-centered).

**Fig 1:**
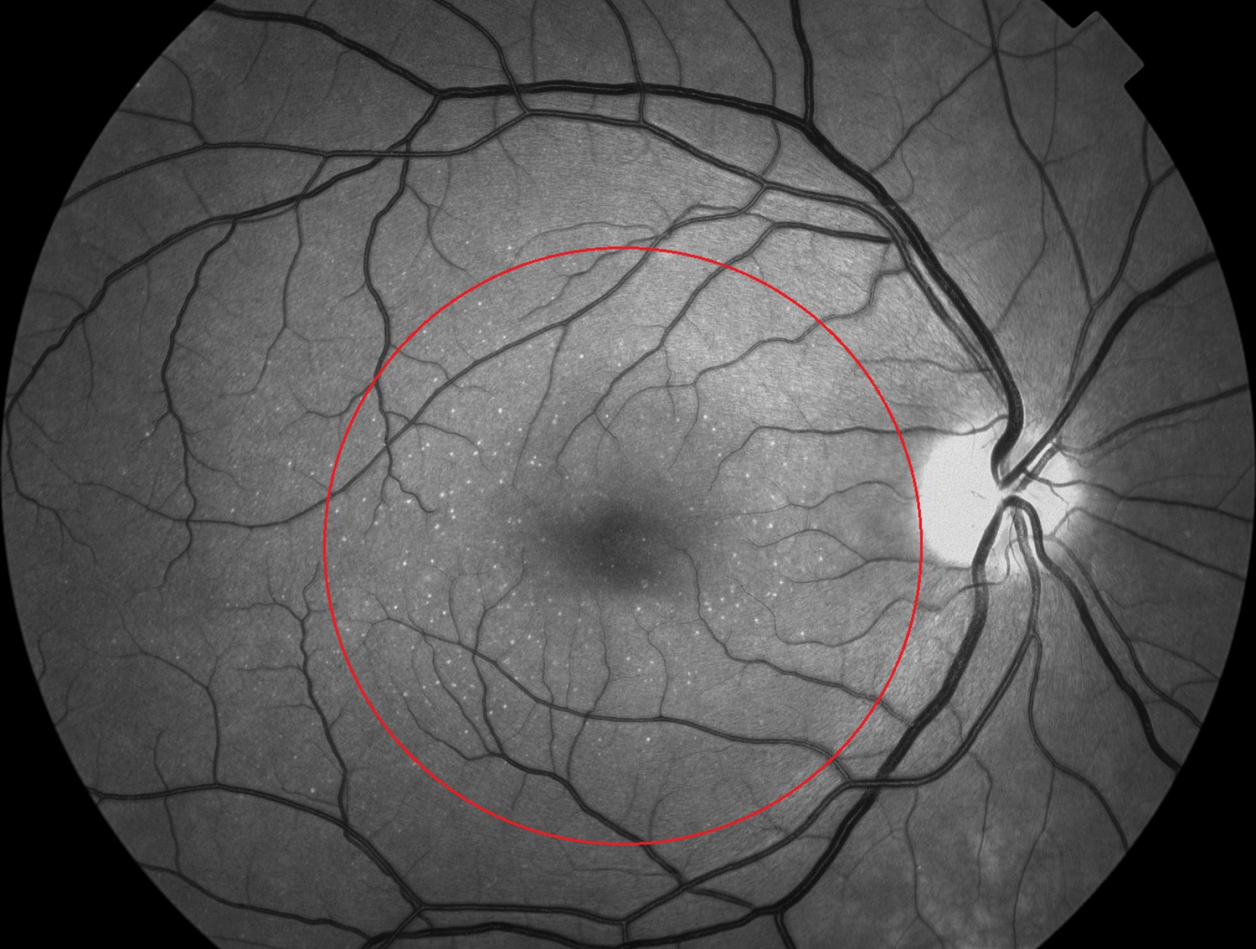
Circle defining the macular field. Red-free fundus photograph of a healthy eye with numerous small hard drusen inside and outside a macular annotation field marked by a red circle.

Fundus images were assessed, graded, and annotated on a digital image platform (Fiji ImageJ v. 1.49 software) by a single observer (MB) masked to age, sex and zygosity. Drusen counts were made on red-free fundus images. Small hard drusen were defined as bright, sharply demarcated elements with a greatest linear diameter < 63 μm. Macular drusen seen on red-free fundus images were reviewed on color fundus images and OCT to help determine their size and category: Small hard drusen (< 63 μm), intermediate drusen (63–125 μm) or large drusen (> 125 μm). Drusen > 125 μm were subcategorized into the following types as seen by OCT: Soft drusen, pachydrusen, and reticular drusen. Cuticular drusen were not observed. Intermediate drusen (63–125 μm) were not reliably classifiable in this system and were regarded as separate entities. Attention was given to avoid confusing drusen-like bright elements with other retinal diseases or conditions, such as hard exudate. Small hard drusen counts (average of the two eyes) were classified as no small hard drusen, 1 to 19 small hard drusen per eye, and 20 or more small hard macular drusen per eye for a macular field defined by a circle centered in the fovea and extending to the temporal edge of the optic nerve head, or 20 or more small hard extramacular drusen per eye for the area outside this circle (Fig 1) [21].

Drusen outside the circle were classified as extramacular drusen. The determination of the number of drusen may be unreliable when over 20 drusen per eye are counted [22]. Therefore, we used ≥ 20 as the threshold for defining numerous small hard drusen. The average number of small hard drusen of a participant’s two eyes was used in all analyses and rounded up to the nearest integer. Analyses of right eyes only and left eyes only gave comparable results. Arbitration of cases, when relevant, was made when relevant by another observer (ML).

Choroidal thickness by SD-OCT was made by manual marking using a dedicated software tool (Heidelberg Eye Explorer, version 1.10.4.0, Heidelberg Engineering, Germany) on a horizontal transfoveal line scan at the center of the fovea and 1 mm temporal of the foveal center.

### Statistical methods

Data analysis was performed using RStudio (Integrated Development Environment for R, Inc., Boston, MA). Appropriate statistical methods were used to correct for paired samples and to calculate outcomes that can be compared with those found in singletons. Comparison of men and women was achieved with a chi-square test for categorical variables and a linear mixed model with fixed effects for continuous variables. The mixed model with fixed effects approach was used to keep various variable estimates constant within twin pairs. Associations between ≥ 20 small hard drusen per eye and potential associations–a priori chosen based on available evidence in the literature–were initially analyzed using a univariate logistical regression model with robust clustered standard errors adjusted for age, sex, and clustering (within pairs of twins). Estimates from the logistical regression model are shown as odds ratio (OR) and 95% confidence interval (CI_95_). For a matched co-twin multivariable analysis, a conditional logistic regression model was used with the same parameters included in the univariate analyses to allow for a more precise estimate of effect measure. Clopper-Pearson confidence interval for a binomial proportion was used for proportional estimates. Age was defined in years and entered as a continuous or ordinal variable (< 49, 50–65, and > 65 years). Estimates of choroidal thickness, spherical equivalent and axial length were comparable for right and left eyes, and this report included an average of both eyes in the regression analyses, respectively. A p-value < 0.05, two-sided, was considered significant.

## Results

The study enrolled 352 participants from 91 monozygotic same-sex twin pairs and 85 dizygotic same-sex twin pairs (Table 1). Five participants were excluded from analysis because the quality of their fundus photographs was insufficient for grading, which left 347 participants for analysis (Table 1). Participant age ranged from 30 to 80 years. All were born in Denmark, and one resided abroad at the time of examination. Examinations were made at the Rigshospitalet.

**Table 1:** Characteristics of the study population.

Variable	All	Men	Women	P-value
No. of participants	347	157	190	
Age (years)	60 (53–65)	60 (52–65)	59 (54–65)	0.94
Body mass index (kg/m^2^)	24.8 (22.8–27.2)	25.2 (23.7–27.1)	24.2 (21.7–27.2)	0.58
Small hard drusen^a^				
No small hard drusen, No. (%)	42 (12.1%)	24 (15.3%)	18 (9.5%)	0.14
Small hard *macular* drusen, n = 1–19 per eye, No. (%) ^a^	262 (75.5%)	119 (75.8%)	143 (75.3%)	1.00
Small hard *macular* drusen, n ≥ 20 per eye, No. (%) ^a^	43 (12.4%)	14 (8.9%)	29 (15.3%)	0.11
Small hard *extramacular* drusen, n ≥ 20 per eye, No. (%) ^a^	96 (27.7%)	28 (17.8%)	68 (35.8%)	0.0003
Intermediate drusen, 63–125 μm, No. (%) ^a^	20 (5.8%)	5 (3.2%)	15 (7.9%)	0.10
Large drusen, > 125 μm, No. (%) ^a^	29 (8.4%)	6 (12.1%)	23 (3.8%)	0.01
Smoking, No. (%)				0.49
No	193 (55.6%)	91 (58.0%)	102 (53.7%)	
Yes	154 (44.4%)	66 (42.0%)	88 (46.3%)	
Alcohol (units per week), No. (%)				0.73
< 7(female) / 14 (male)	278 (80.1%)	124 (79.0%)	154 (81.1%)	
> 7(female) / 14 (male)	69 (19.9%)	33 (21.0%)	36 (18.9%)	
Diabetes, No. (%)				0.53
No	335 (96.5%)	150 (95.5%)	185 (97.4%)	
Yes	12 (3.5%)	7 (4.5%)	5 (2.6%)	
Mean arterial pressure, mmHg	103 (95–112)	103 (97–113)	103 (95–112)	0.15
Serum total cholesterol, mmol/L	5.30 (4.70–6.00)	5.20 (4.50–5.90)	5.50 (4.90–6.10)	0.02
Serum LDL cholesterol, mmol/L	3.10 (2.40–3.60)	3.10 (2.40–3.60)	3.10 (2.48–3.60)	0.77
Serum HDL cholesterol, mmol/L	1.54 (1.26–1.96)	1.32 (1.14–1.60)	1.76 (1.42–2.21)	< 0.0001
Serum triglycerides, mmol/L	1.36 (0.97–1.82)	1.47 (1.09–2.00)	1.26 (0.92–1.69)	0.003
Spherical equivalent	0.00 (-1.00–1.00)	0.00 (-1.00–1.00)	0.00 (-0.92–1.00)	0.77
Axial length, mm	23.7 (23.1–24.4)	23.9 (23.3–24.6)	23.5 (22.8 24.0)	0.0004
Subfoveal choroidal thickness, μm	319 (253–386)	313 (251–386)	322 (258–385)	0.53

Estimates are median and interquartile range unless otherwise stated. A chi-squared test was computed for a categorical variable, and for a continuous variable, a linear mixed regression model with fixed effects to adjust for clustering (twins) was used. An average of both eyes was used for spherical equivalent, axial length, and choroidal thickness estimates.

^a^An average number of both eyes was used to calculate total number of small hard *macular* drusen. Small hard *extramacular* drusen were stipulated independent of small hard *macular* drusen; hence, a participant can have both ≥ 20 *macular* and ≥ 20 *extramacular* small drusen. Intermediate and large soft drusen were considered as binary variables (yes / no) independent of small hard drusen.

LDL = low-density lipoprotein; HDL = high-density lipoprotein.

Small hard macular drusen were found in 271 right eyes (78.1% [CI_95_ 73.4% - 82.3%]) and 254 left eyes (73.1% [CI_95_ 68.2% - 77.8%]). One or more small hard macular drusen in either eye were found in 305 participants (87.9% [CI_95_ 84.0% - 91.1%]). The distribution was comparable in men and women (p = 0.14, chi-square test). Recounts of small hard macular drusen showed an intraclass correlation coefficient for conformity among the counts vs. recounts of 0.98 [CI_95_ 0.98–0.99], p < 0.001, for right eyes with identical numbers for left eyes.

The characteristic ≥ 20 small hard macular drusen per eye was found in 43 (12.4%) participants, and the phenotype ≥ 20 small hard extramacular drusen per eye was found in 96 (27.7%). The characteristic ≥ 20 small hard macular drusen per eye combined with ≥ 20 small hard extramacular drusen per eye was found in 33 (9.5% of the total study population). Intermediate drusen were found in 20 (5.8%) participants, large drusen were found in 29 (8.4%), and only two participants had late-stage AMD.

**Fig 2:**
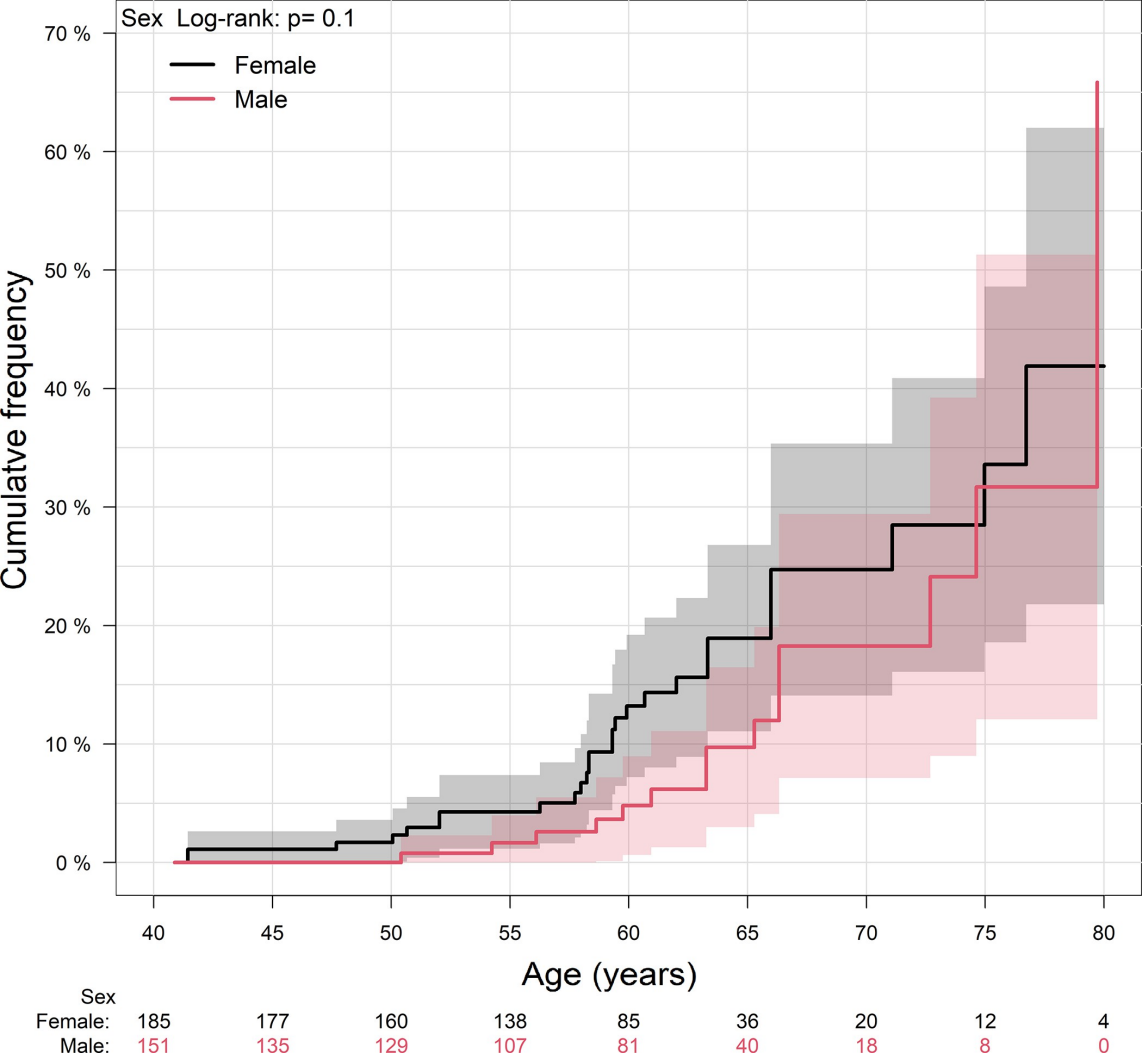
Cumulative frequency of numerous small hard macular drusen. Cumulative frequency of having ≥ 20 small hard macular drusen per eye as a function of age and sex.

The proportion of participants with 1–19 drusen per eye decreased with each increase in age bracket in proportion to a concomitant rise in the proportions of participants with ≥ 20 small hard macular drusen and participants with drusen ≥ 63 μm (Table 2, Fig 2). This trajectory did not differ between men and women.

**Table 2:** Per-participant distribution of macular drusen based on size and pigmentation by age group.

Age	No.	Small hard drusen			Pigmentation	Intermediate drusen, 63–125 μm	Large drusen, > 125 μm
		0 per eye	1–19 per eye	≥ 20 per eye			
< 49 y	58	6 (10.3%)	49 (84.5%)	3 (5.2%)	0	2 (3.4%)	0
50–65 y	231	22 (9.5%)	180 (77.9%)	29 (12.6%)	5 (2.2%)	14 (6.1%)	18 (7.8%)
> 65 y	58	14 (24.1%)	33 (56.9%)	11 (19.0%)	2 (3.4%)	4 (6.9%)	11 (19.0%)
Total	347	42 (12.1%)	262 (75.5%)	43 (12.4%)	7 (2.0%)	20 (5.8%)	29 (8.4%)
P-value		0.009	0.001	0.08	0.40	0.69	0.001

The classification was based on the averages of the participants’ two eyes. The primary classification is non-hierarchical, i.e., a given participant is represented in all classes. However, for pigment and drusen ≥ 63 μm, the characteristic of having none of these is evident only in participants not being listed as having them. The secondary classification by the number of small hard drusen per eye permits participation in only one out of the three subclasses. Pearson’s chi-square test was applied for a column-wise p-value comparing each age group within each phenotype category. The parentheses show the fraction of affected participants within each age bracket.

In the univariate analyses, the odds of having ≥ 20 hard macular drusen per eye increased significantly with age (p = 0.04, Table 3). A trend was seen for a higher prevalence of ≥ 20 hard macular drusen per eye and lower body mass index (BMI). In a univariate analysis, the trait ≥ 20 small hard extramacular drusen per eye was associated with increasing age, female sex, lower BMI, shorter axial length, and a thicker choroidal (Table 3).

**Table 3:** Age-, sex-, and cluster-adjusted generalized linear model of potential associations for the univariate outcomes ≥ 20 hard *macular* drusen per eye and ≥ 20 hard *extramacular* drusen per eye.

Variable	*Macular*, small hard drusen n ≥ 20, odds ratio [CI_95_]	P-value	*Extramacular*, small hard drusen, n ≥ 20, odds ratio [CI_95_]	P-value
Age, per 5-year increase	1.22 [1.03–1.46]	0.04	1.36 [1.17–1.53]	< 0.0001
Women	1.87 [0.87–4.01]	0.11	2.76 [1.44–5.28]	0.002
Body mass index, kg/m^2^	0.88 [0.79–1.00]	0.05	0.88 [0.82–0.94]	< 0.0001
Smoking				
No	1		1	
Yes	1.37 [0.66–2.85]	0.39	0.95 [0.54–1.66]	0.86
Alcohol				
< 7 (females) / 14 (males)	1		1	
> 7 (females) / 14 (males)	0.76 [0.34–1.71]	0.51	1.93 [0.98–3.80]	0.06
Serum HbA_1c_, mmol/mol	0.97 [0.87–1.08]	0.53	0.96 [0.90–1.03]	0.28
Mean arterial blood pressure, per 5 mmHg increase^a^	1.01 [0.98–1.04]	0.61	0.94 [0.85–1.05]	0.24
Serum total cholesterol, mmol/L	0.93 [0.68–1.27]	0.63	0.87 [0.66–1.14]	0.31
Serum LDL cholesterol, mmol/L	0.95 [0.65–1.39]	0.80	0.82 [0.60–1.13]	0.23
Serum HDL cholesterol, mmol/L	0.93 [0.43–1.99]	0.85	1.21 [0.63–2.31]	0.57
Serum triglycerides, mmol/L	1.00 [0.57–1.77]	0.99	0.88 [0.56–1.37]	0.56
Axial length, mm	0.75 [0.55–1.00]	0.05	0.62 [0.48–0.80]	0.0002
Spherical equivalent, diopter	1.08 [0.93–1.26]	0.31	1.16 [1.04–1.30]	0.01
Subfoveal choroidal thickness, per 50 μm increase	1.10 [0.93–1.31]	0.28	1.22 [1.06–1.39]	0.004

Comorbidity variables heart disease, respiratory disease, gastrointestinal disease and neurological disease were omitted due to too few cases. For continuous variables, odds ratio estimates have been calculated per one-unit increase unless otherwise indicated. An average of both eyes was used for spherical equivalent, axial length, and choroidal thickness estimates.

^a^Participants diagnosed with hypertension have been censored.

CI_95_ = 95% confidence interval; LDL = low-density lipoprotein; HDL = high-density lipoprotein.

In multivariable analysis (Table 4), the odds of having ≥ 20 small hard macular drusen per eye also increased with age, lower BMI, and shorter axial length. The odds of having ≥ 20 small hard extramacular drusen increased with age, female sex, lower BMI, high alcohol consumption, high HDL, hyperopia, and shorter axial length (Table 4). The effect of a thicker choroid disappeared when the parameter was analyzed in conjunction with axial length and spherical equivalent refraction.

**Table 4:** Multivariable conditional logistical regression model of variables associated with ≥ 20 small hard *macular* drusen per eye and ≥ 20 hard *extramacular* drusen per eye.

Variable	*Macular*, small hard drusen, n ≥ 20, odds ratio [CI_95_]	P-value	*Extramacular*, small hard drusen, n ≥ 20, odds ratio [CI_95_]	P-value
Age, per 5-year increase	1.21 [1.02–1.44]	0.02	1.33 [1.17–1.52]	< 0.0001
Women	1.92 [0.97–3.81]	0.06	1.79 [1.14–2.82]	< 0.01
Body mass index, kg/m^2^	0.88 [0.79–0.97]	0.01	0.91 [0.85–0.96]	0.003
Smoking				
No	1		1	
Yes	1.37 [0.72–2.60]	0.33	1.05 [0.67–1.63]	0.84
Alcohol				
< 7(female) / 14 (male)	1		1	
> 7(female) / 14 (male)	0.77 [0.33–1.82]	0.56	1.80 [1.05–3.10]	0.04
Mean arterial pressure, per 5 mmHg increase^a^	1.01 [0.99–1.04]	0.30	1.00 [0.98–1.02]	0.76
HbA_1c_, mmol/mol	1.00 [0.94–1.05]	0.86	1.00 [0.96–1.04]	0.91
Serum total cholesterol, mmol/L	1.04 [0.74–1.46]	0.82	0.97 [0.77–1.22]	0.80
Serum LDL cholesterol, mmol/L	1.00 [0.68–1.48]	0.99	0.90 [0.68–1.17]	0.42
Serum HDL cholesterol, mmol/L	1.28 [0.67–2.45]	0.46	1.67 [1.05–2.42]	0.04
Serum triglycerides, mmol/L	0.99 [0.65–1.51]	0.97	0.79 [0.59–1.07]	0.13
Spherical equivalent, diopter	1.12 [0.97–1.29]	0.12	1.22 [1.09–1.36]	< 0.0001
Axial length, mm	0.72 [0.53–0.97]	0.03	0.62 [0.50–0.77]	< 0.0001
Subfoveal choroidal thickness, per 50 μm increase	1.04 [0.88–1.22]	0.56	1.10 [0.98–1.23]	0.10

A conditional logistic model was used for a matched co-twin design; all variables were computed as covariables. Relevant variables from the univariate analysis were entered into the multivariable analysis. For continuous variables, odds ratio estimates have been calculated per one-unit increase unless otherwise indicated. An average of both eyes was used for spherical equivalent, axial length, and choroidal thickness estimates.

^a^Participants diagnosed with hypertension have been censored.

CI_95_ = 95% confidence interval; LDL = low-density lipoprotein; HDL = high-density lipoprotein.

Large drusen (> 125 μm) were associated with ≥ 20 small hard macular drusen per eye (p = 0.02, chi-square test) but not with ≥ 20 small hard extramacular drusen per eye (Table 5).

**Table 5:** Distribution of large drusen (> 125 μm) subtypes among participants with ≥ 20 or < 20 small hard drusen.

Small hard drusen subgroup		Drusen (> 125 μm) subtypes				P-value
		None	Soft drusen	Pachydrusen	Reticular drusen	
Small hard *macular* drusen per eye	No. of n < 20 (%)	282 (92.8%)	5 (1.6%)	16 (5.3%)	1 (0.3%)	0.02
	No. of n ≥ 20 (%)	36 (83.7%)	5 (11.6%)	1 (2.3%)	1 (2.3%)	
Small hard *extramacular* drusen per eye	No. of n < 20 (%)	288 (94.7%)	5 (2.0%)	9 (3.6%)	2 (0.8%)	0.41
	No. of n ≥ 20 (%)	83 (86.5%)	5 (5.2%)	8 (8.3%)	0	

Pearson’s chi-square test was applied for a row-wise p-value. Using a one-way ANOVA test, a comparison of age differences between large drusen subtypes showed no statistical difference (p = 0.18). The classification of small hard drusen subgroups is non-hierarchical; a participant can figure in both classes.

The median number of small hard macular drusen was 9 (interquartile range [IQR] = 4–24) in participants with large drusen and 3 (IQR = 1–8) in participants without large drusen (p = 0.008).

The median number of small hard extramacular drusen was 15 (IQR = 6–56) in participants with large drusen and 6 (IQR = 2–21) in participants without large drusen (p = 0.24).

## Discussion

This study of small hard drusen in people in the early years of AMD age range found that 12.4% of participants had the prominent characteristic of having ≥ 20 small hard macular drusen per eye. Small hard drusen are not a part of the AMD fundus lesion spectrum, as it is currently defined, but the trait was found to share certain risk factors with AMD, including higher age, shorter axial length, and hyperopia. An effect of a thicker choroid sank below the level of statistical significance after correction of refraction and axial length. However, the three factors are closely correlated, with the choroidal thickness being the factor that is by far the most difficult to measure, suggesting that length and refraction may be surrogate measures of a mechanistic factor that resides in the choroid.

Small hard drusen were correlated with the presence of large drusen, i.e., drusen with a diameter greater than 125 μm. Because no large drusen were found in 1999, when one-half of the participants were seen, the most obvious interpretation is that ≥ 20 small hard macular drusen per eye are a risk factor for developing large drusen, as previously shown [5,6].

The Watermen Study showed that five or more small hard drusen were associated with an increased 5-year incidence of large drusen [5]. The Beaver Dam Eye Study showed that the larger the posterior pole area covered with small hard drusen at baseline, the higher the 15-year cumulative incidence of early AMD [6]. The progression from small hard drusen to the larger drusen compatible with the diagnosis of AMD in the aforementioned studies was based on eye-by-eye grading, not in situ lesion-by-lesion comparisons. Thus, small hard drusen can be seen to disappear [1], but the direct progression from small hard drusen to large drusen in the same location has only recently been explored by our study group with our twin cohort in a 20-year follow-up study. We identified three participants where large drusen had developed in a place previously occupied by small hard drusen. Also, having ≥ 20 small hard drusen at baseline increased the 20-year risk of developing larger drusen [4].

Clinicopathological correlation between small hard drusen in vivo and in histopathology sections has been made on a small scale [1,2]. These findings suggest that small hard drusen may be part of a continuum encompassing drusen ≥ 63 μm and hence fit the diagnosis of AMD.

If small hard drusen are indeed precursors or heralds of larger soft drusen, one would assume similar risk profiles in terms of modifiable and non-modifiable risk factors between the two phenotypes, which was not the case in this study. This raises the suspicion that small hard drusen may be a selective precursor for some but not all types of drusen compatible with the diagnosis of AMD.

The close resemblance of the risk factors for small hard drusen inside and outside the macula warrants consideration of whether the two can be merged in natural history studies. The larger total drusen numbers would help justify their treatment as a continuous variable in statistical analyses.

This study found that longer axial lengths decreased the odds of having ≥ 20 small hard macular or ≥ 20 small hard extramacular drusen. This agrees with studies that found a higher incidence of AMD in shorter eyes (axial length < 23 mm) [14,23]. A shorter axial length is strongly associated with a thicker choroid [24] and hyperopia, all of which were associated with ≥ 20 small hard extramacular drusen in the present study and, in another study, with a higher risk of incident AMD [13]. In the AREDS cohort, the authors also found an association between hyperopia and late AMD [25]. The close correlation between axial length, refraction, and choroidal thickness may explain why no independent effect was found of the latter in the present study (Table 4). Thus, the Copenhagen Child Cohort 2000 Study of healthy children found that a thicker choroid was associated with higher odds of having small hard drusen [26]. A thicker choroid is associated with pachydrusen [27]. Theoretically, stretching of Bruch’s membrane in myopia may increase its pore size, reduce its thickness, and promote increased flux through the membrane, thus reducing the formation of drusen.

Higher HDL and female sex were associated with ≥ 20 extramacular drusen. Similar results were found in a large review examining AMD risk factors, although some of the included studies found no associations between higher HDL or female sex and increased AMD risk [28].

Higher body mass index was associated with lower odds for having ≥ 20 small hard macular and extramacular drusen, which is in contrast to AMD, where excess body weight was associated, in a dose-dependent, with AMD [29]. Furthermore, alcohol overconsumption was associated with having ≥ 20 small hard extramacular drusen. In contrast, a systematic review and meta-analysis found no relationship between alcohol intake and AMD [30].

Smoking is of particular interest among the factors known to influence AMD that had no measurable effect on small hard drusen in this study. Smoking is a strong promotor of AMD [31]. The lack of association with small hard drusen confirms our previous finding in the Inter99 Eye Study [21]. Also, higher arterial blood pressure was not associated with ≥ 20 small hard drusen, macular or extramacular, unlike AMD, which is associated with hypertension [25]. However, a limitation regarding hypertension in the present study should be noted, namely that we used mean arterial pressure as the descriptive variable for blood pressure.

The study cohort covered an age spectrum that suits the study of early AMD. Thus, only two participants had late AMD. The number of participants with ≥ 20 small hard drusen per eye (macular, n = 43 and extramacular, n = 96) was sufficient, however, to identify a risk factor profile for small hard drusen. It was found to differ significantly from that of AMD. Thus, the study provides new information about a stage in the transition from not having AMD to having AMD and highlights the role of potential precursor lesions for drusen compatible with the diagnosis of AMD. Limitations of the study includes the reliance on self-reporting of body weight, tobacco, and alcohol consumption, which entails the risk of recency and recall bias. Although we adjusted for dependencies between twins, there are both data limitations and imperfections of statistical models. Finally, genotypes, dietary habits, and physical activity were not examined or acquired.

In conclusion, we observed that certain AMD-associated risk factors were associated with the presence of ≥ 20 small hard macular drusen: increasing age, shorter axial length, hyperopia, and female sex. Unlike AMD, having ≥ 20 small hard macular drusen was not associated with alcohol consumption, smoking, higher blood pressure, and dyslipidemia. The study results may help advance the study of drusen formation and drusen subtypes and assist the development of early interventions against AMD.
